# Integrated transcriptomics and metabolomics analysis provide insight into anthocyanin biosynthesis for sepal color formation in *Heptacodium miconioides*

**DOI:** 10.3389/fpls.2023.1044581

**Published:** 2023-02-20

**Authors:** Yueling Li, Zhongshuai Sun, Jieyang Lu, Zexin Jin, Junmin Li

**Affiliations:** ^1^ Zhejiang Provincial Key Laboratory of Plant Evolutionary Ecology and Conservation, Taizhou, China; ^2^ Institute of Ecology, Taizhou University, Taizhou, China

**Keywords:** Heptacodium miconioides, sepal development, transcriptomic, metabolomic, anthocyanin

## Abstract

*Heptacodium miconioides* Rehd., commonly known as “seven-son flower,” is an ornamental species with a beautiful flower pattern and persistent sepals. Its sepals are of horticultural value, turning bright red and elongating in the autumn; however, the molecular mechanisms that cause sepal color change remain unclear. We analyzed the dynamic changes in anthocyanin composition in the sepal of *H. miconioides* at four developmental stages (S1-S4). A total of 41 anthocyanins were detected and classified into 7 major anthocyanin aglycones. High levels of the pigments cyanidin-3,5-O-diglucoside, cyanidin-3-O-galactoside, cyanidin-3-O-glucoside, and pelargonidin-3-O-glucoside were responsible for sepal reddening. Transcriptome analysis revealed 15 differentially expressed genes involved in anthocyanin biosynthesis that were detected between 2 developmental stages. Of these, the high expression of *HmANS* was considered critical structural gene related to anthocyanin biosynthesis pathway in the sepal through co-expression analysis with anthocyanin content. In addition, a transcription factor (TF)-metabolite correlation analysis revealed that three HmMYB, two HmbHLH, two HmWRKY, and two HmNAC TFs exhibited a strong positive role in the regulation of the anthocyanin structural genes (Pearson’s correlation coefficient > 0.90). Luciferase activity assay showed that HmMYB114, HmbHLH130, HmWRKY6, and HmNAC1 could activate the promoters of *HmCHS4* and *HmDFR1* genes *in vitro*. These findings increase our understanding of anthocyanin metabolism in the sepal of *H. miconioides* and provide a guide for studies involving sepal color conversion and regulation.

## Introduction

Anthocyanin is classified as a flavonoid and is present in land plants. To date, at least 635 anthocyanins have been identified in nature (He and Giusti, 2010). Six categories of anthocyanins were the most common in the blue, red, and purple colors of plants, including pelargonidin, cyanidin, delphinidin, peonidin, petunidin, and malvidin (Tanaka et al., 2008; Yang et al., 2015). In flowering plants, the color changes that occur in floral organs are closely related to anthocyanins (Shen et al., 2021). Thus far, several genes involved in the anthocyanin biosynthesis pathway have been identified in most plants (Boss et al., 1996; Saito et al., 2013; Zheng et al., 2022). These genes mainly include dihydroflavonol 4-reductase (DFR), anthocyanidin synthase (ANS), and UDP-glucose: flavonoid 3-glucosyltransferase (UFGT) (Schaart et al., 2013; Wang et al., 2019). Subsequently, anthocyanins are accumulated and stored through anthocyanin glycosylation, glutathione S- transferase (GST) proteins, multidrug and toxic compound extrusion (MATE) transporters, etc. (Koes et al., 2005). A protein complex composed of MYB TF and bHLH regulator together with a WD40 repeat protein (MYB-bHLH-WD40, MBW) could binds to the promoters of anthocyanin structural genes to activate their expression (Ramsay and Glover, 2005). For example, Carretero-Paulet et al. (2010) showed that MYB123/bHLH42/TTG1 and MYB75/bHLH2/TTG1 are involved in the expression of anthocyanin biosynthesis genes in *Arabidopsis thaliana*. In addition, other TFs, such as WRKY72, NAC52, HY5 and ERF1B have also been identified to be directly or indirectly involved in the biosynthesis of anthocyanin (An et al., 2017; Zhang et al., 2018; Sun et al., 2019; Hu et al., 2020). The sepals, which play an important role in protecting the other floral organs (stamens, pistils, and petals), generally surround the outside of the flower. In some plants, sepals are uniformly green and provide the energy required for fruit or seed development through photosynthesis (Liu et al., 2020; Li et al., 2020a). In other plants, such as *Hydrangea macrophylla*, the sepals perform the function of petals. The actual flower petals are replaced by sepals that have the ability to change color by accumulating anthocyanins (Yoshida et al., 2021). Whether the anthocyanins in sepals and their biosynthetic pathways are the same as that in the petals is unclear. To date, little is known regarding the underlying mechanism of color formation in sepals.

The Caprifoliaceae family contains more than 800 species, some containing pink or white flowers with fragrance and persistent sepals, which have a long duration and ornamental period. A representative species of this family, *Heptacodium miconioides* Rehd., which is commonly known as “seven-son flower” in China and is a deciduous tree from the *Heptacodium* genus (Coombes, 1990; Bian et al., 2002; Zhang et al., 2022). Although this plant is native to China, it is now widely cultivated in botanical institutions and nurseries throughout the world (Coombes, 1990). The tree is elegant in shape and has beautiful white flowers with a jasmine-like scent. The sepals of its flowers enlarge and change color from green to red, as if undergoing a second blooming. Its beauty has attracted the attention of many horticulturalists (Liu et al., 2006). Therefore, identifying the mechanism of sepal color formation will be valuable for understanding plant evolution and for breeding novel ornamental lines. Currently, there are few systematic studies on the characteristics and coloration of *H. miconioides* sepals (Liu et al., 2006).

Recently, transcriptomics and metabolomics have been widely used to explore the relationships between genes and metabolites, and to unravel structural genes and TFs that may play a role in secondary metabolic pathways (Dong et al, 2009; Wu et al., 2020). For example, in the flowers of tea (*Camellia sinensis* L.), the *DFR*, *CHS*, *F3H*, *FLS*, and *LDOX1* genes at five developmental stages were identified by metabolomics and transcriptome sequencing analysis. These genes were found to have a direct relationship with the biosynthesis and accumulation of cyanidin 3-O-glucoside and petunidin 3-O-glucoside (Rothenberg et al., 2019). Chen et al. (2022) performed a metabolome and transcriptome analysis of sweet cherry (*Prunus avium* L.), which revealed a different temporal expression pattern of anthocyanin accumulation associated with fruit color. They suggested that the MYC2, NAC71, WRKY57, and TCP7 TFs might be participated in the regulation of anthocyanin structural genes. Similarly, Zheng et al. (2022) found that the *UFGT* gene and two *ANS* genes were upregulated in the whole developmental stages of *Zanthoxylum bungeanum* fruit peels, which may represent key functional genes for the accumulation of peonidin 3-O-glucoside and peonidin O-hexoside. Therefore, multiomics analysis is a powerful strategy to explore the regulation of the anthocyanin network at different developmental stages in plants (Liu et al., 2017; Lin et al., 2020).

In this study, we determined the differences in anthocyanin biosynthesis during *H. miconioides* sepal development. Candidate genes and regulators of anthocyanin biosynthesis were identified using a transcriptomic analysis. The anthocyanin compounds in the different sepal developmental stages were determined using multiple reaction monitoring (MRM). Then, an unbiased network analysis was constructed to detect the relationship between genes and anthocyanin accumulation. Our major objectives were (1) to elucidate the potential key genes of enzyme and TFs involved in the anthocyanin biosynthesis pathway at different sepal developmental stages, and (2) to explore the potential regulatory mechanisms underlying the biosynthesis of anthocyanin in the sepal of *H. miconioides*. The results provide insight into anthocyanin biosynthesis in the sepal of this ornamental species and increase our understanding of the molecular mechanisms responsible for sepal color development.

## Materials and methods

### Plant materials

The *H. miconioides* used in this study was cultivated at the Taizhou University botanical garden, Zhejiang Province, China (121°17′E, 28°87′N; 10 m above sea level) under natural conditions. The annual average of temperature and precipitation is approximately 17.10°C and 1,231.40 mm, respectively. The growth soil condition of *H. miconioides* is mainly yellow loam with a pH of approximately 6.10. Sepal samples from healthy plants grown under natural light were collected from 9:00 to 11:00 in September to October 2018. Sepal samples were collected during four sepal developmental stages, corresponding to lengths of 0.1 cm (S1), 0.3 cm (S2), 0.5 cm (S3), and 0.8 cm (S4), respectively. The samples were frozen in liquid nitrogen immediately after collection and stored at −80°C for further transcriptomic and metabolomic analysis. In addition, to ensure the consistency of the subsequent correlation analysis, samples representing each period were mixed and divided into two parts, which were used for transcriptome sequencing and metabolite analysis. There were three biological replicates for each sepal from each period for a total of 12 samples.

### Anthocyanin measurement

Anthocyanin content was analyzed as previously described, with minor modifications (Lin et al., 2020). Briefly, sepal samples were freeze-dried and powdered in a MM 400 grinder (Retsh Technology, Germany). Then, 50 mg of powdered samples were extracted with 500 μL of 0.1% (v/v) hydrochloric methanol solution for 20 h at 4°C. The pigment extract sample was filtered through an HPLC PTFE syringe filter (0.22 μm). The anthocyanin composition was analyzed using UPLC (ExionLC™ AD) equipped with a reverse Waters ACQUITY BEH C18 column (1.7 µm, 2.1 mm*100 mm) and Tandem mass spectrometry (MS/MS, Applied Biosystems 6500 QTRAP). The UPLC analysis was performed under the following conditions: solvent system, ultrapure water (0.1% formic acid): methanol (0.1% formic acid); gradient program, 95:5 v/v at 0 min, 50:50 v/v at 6.0 min, 5:95 v/v at 2.0 min, and 95:5 v/v at 14.0 min; flow rate, 0.35 mL/min; temperature, 40°C; injection volume, 2 μL. Based on the MetWare database (MetWare Biotechnology Co., Ltd., Wuhan, China), the data detected by mass spectrometry was analyzed qualitatively. The concentration of anthocyanin in the sample was calculated using MRM. The MRM for each sepal sample was measured in triplicate. A metabolite heatmap was generated by complexheatmap R package (version 2.7.1.1009) after unit variance scaling. Differentially accumulated metabolites (DAMs) were identified based on a log_2_(FoldChange) ≤ 0.5 or ≥2 and a variable importance in the projection (VIP) ≥1.

### Transciptome analysis

The sepal samples at four developmental stages used for transcriptomics were the same as the anthocyanin analysis samples. The genome of *H. miconioides* was used as a reference (Unpublished data). Functional annotation information for all expressed genes was obtained from the *H. miconioides* genome annotation project using the following public databases: Nr (http://www.ncbi.nlm.nih.gov/protein ), SwissProt (http://www.uniprot.org/ ), Pfam (http://pfam.xfam.org/ ), InterPro (https://www.ebi.ac.uk/interpro/ ) and KEGG (http://www.genome.jp/kegg/ ), with an e-value threshold of 1e^-05^. We also confirmed the identified candidate genes involved in anthocyanin biosynthesis using knowledge-based pathway enzyme identification (KIPEs, version 3) (Pucker et al., 2020). Total RNA was extracted from the four stages of sepal development with three biological replicates. Libraries were prepared and sequenced using the Illumina HiSeq 2500 platform (Illumina, San Diego, CA, USA) and paired-end reads were generated. The raw paired-end RNA-Seq reads were filtered into clean data using FASTP (version 0.19.5) (Chen et al., 2018). The RNA clean reads were aligned to the reference genome using HISAT2 (version 2.0.4) (Kim et al., 2015). The FPKM value was used to calculate gene expression levels. DESeq (version 1.10.1) with a model based on the negative binomial distribution was used to identify differentially expressed genes (DEGs) (Anders and Huber, 2010), and the screening threshold was set as *p*adj < 0.05 and the absolute value of log_2_(FoldChange) ≥1 between different developmental stages (S1 vs. S2, S2 vs. S3, S3 vs. S4, S1 vs. S3, S1 vs. S4, and S2 vs. S4).

### Analysis of TFs

The identification of the MYB TF was based on the freely available described in a previous study (https://github.com/bpucker/MYB_annotator , version 0.23) (Pucker, 2022). The bHLHs, WRKYs, NACs, bZIPs, and HSFs TFs families from the transcriptome data were predicted with iTAK software (version 1.2) (Zheng et al., 2016). The parameters of iTAK were set as follows: -s n -m b -a 5, where -s is sequence type and nucleic acid sequence, -m is the type of analysis, and -a is a hmmscan computational resource. The TF homologous protein sequences of *A. thaliana* that were downloaded from the TAIR database. The phylogenetic tree of the predicted TFs and the known TFs in *A. thaliana* was generated by the MEGA version 11.0.13 program (Tamura et al., 2021) using the neighbor-joining method with default parameters (1,000 bootstrap replicates). Phylogenetic trees were modified using iTOL (https://itol.embl.de/personal_page.cgi ). *H. miconioides* TFs homologs were classified on the basis of their relationships with corresponding *A. thaliana*.

### Combined transcriptome and metabolome analysis

To determine potential key candidate structural genes and TFs involved in anthocyanin biosynthesis pathway in *H. miconioides*, a joint analysis between transcriptomic and metabolomic datasets was performed. Joint analysis parameters were established by calculating the mean of three biological replicates of the DEGs, differential transcript expression FPKM value of the transcriptomic data, and the mean of the differential anthocyanin components in metabolomic data. The resulting data was log transformed with R (www.r-project.org/ ) and a correlation analysis between differential genes and metabolites was conducted by calculating pearson correlation coefficient (PCC) values in hmisc R package (4.4.0). Significant correlations were considered at **|**PCC**|** > 0.80 and *p*-value < 0.05 (Lin et al., 2020). The network diagrams were visualized by Cytoscape software (version 3.6.1).

### Transient activation assays in *Nicotiana benthamiana* leaves

The CDS fragments of HmMYB114, HmMYB11, HmbHLH130, HmWRKY6, HmNAC1, and HmNAC2 was cloned into the pCAMBIA1300-35S vector (effector) (pro35S:HmMYB114; pro35S:HmMYB11; pro35S:HmbHLH130; pro35S:HmWRKY6; pro35S:HmNAC1; pro35S:HmNAC2). The promoter sequences of *HmCHS4* and *HmDFR1* were amplified from the genomic DNA and then transformed into the pLLOOR-Bar luciferase (LUC) vector (reporter) (producing proHmCHS4:LUC). *Agrobacterium tumefaciens* GV3101 harbouring the effector and reporter vectors was injected into *N. benthamiana* leaves following the method described in Lou et al., 2018. The LUC activity tests were performed using a Tanon-5500 chemiluminescence imaging system (Tanon, Shanghai, China) according to the manufacturer’s instructions. The LUC signal intensity value was generated by TanonImage (version 201201 1.10) with a quantitation parameter of 1ng standard. The single *HmCHS4* and *HmDFR1* promoter-LUC recombinant vector was used as the blank control. The experiments were technically repeated three times with similar results. The primer sequences for the LUC assays involved in anthocyanin biosynthesis genes are listed in **Supplementary Table 1**.

### Statistical analysis

The data was analyzed using SPSS 20.0 statistical software. A one-way analysis of variance was used to test the differences of total anthocyanin contents at different sepal developmental stages and relative LUC signal intensity, followed by LSD or Dunnett’s T_3_ test with significant differences at 5% level. Origin 8.5 software was used to draw graphs. The data are presented as the mean ± standard deviations (SD).

## Results

### Total anthocyanin content associated with sepal of *H. miconioides*

**Figure 1:**
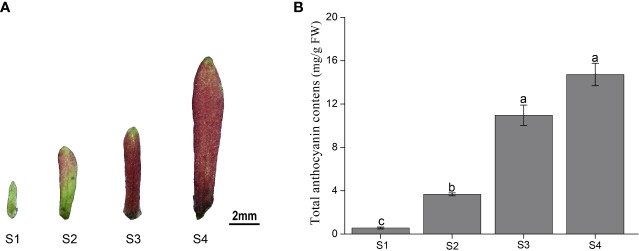
Color and total anthocyanin content of *Heptacodium miconioides* sepals at four developmental stages (S1-S4). **(A)** The characteristics of the (*H*) *miconioides* sepal at four growth stages. **(B)** Changes in total anthocyanin content during sepal development. S1, sepal length = 0.1 cm; S2, sepal length = 0.3 cm; S3, sepal length = 0.5 cm; S4, sepal length=0.8 cm. Values are the means ± standard deviation (SD). Data is the mean of three biological replicates. Different lowercase letters indicated significant differences at 0.05 level.

The color of the sepals of *H. miconioides* changed from green to dark red at the different developmental stages (**Figure 1A**). To gain insight into the differences in anthocyanin compounds and biosynthesis, four sepal samples were used for anthocyanin-targeted metabolome analysis. The results indicated that the total anthocyanin content gradually increased during sepal development. The total anthocyanin content of the sepals in S3 and S4 was significantly higher compared with that in S1 and S2, but there was no significant difference between S3 and S4 (**Figure 1B**).

### Anthocyanin metabolites in the sepals of *H. miconioides*

**Figure 2:**
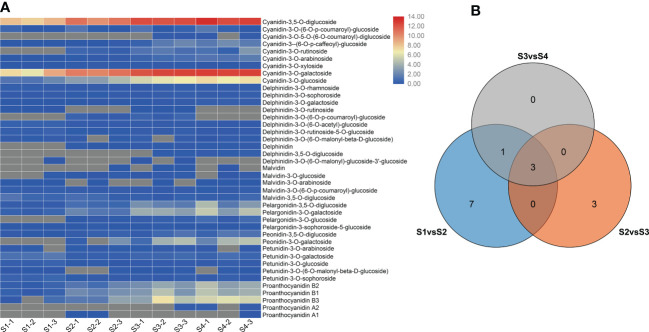
Differential anthocyanin metabolite content in *Heptacodium miconioides* sepals during development. **(A)** Heat maps of DAMs in the S1 through S4 developmental stages. **(B)** Venn diagram of DAMs shared among two or three comparisons.

To compare the differences in anthocyanin metabolite content between the S1 to S4 sepals of *H. miconioides*, we used UPLC/ESI-QTRAP-MS/MS. A total of 41 anthocyanin metabolites were detected in the sepal samples of *H. miconioides* (**Figure 2A** and **Supplementary Table 2**). The 41 anthocyanin metabolites were classified into 7 categories: cyanidin, delphinidin, malvidin, pelargonidin, peonidin, petunidin, and proanthocyanidin. Of these, the main components of anthocyanin content were cyanidin-3,5-O-diglucoside, cyanidin-3-O-galactoside, cyanidin-3-O-glucoside, proanthocyanidin B3, pelargonidin-3-O-glucoside, and peonidin-3-O-galactoside (**Figure 2A** and **Supplementary Table 2**). There were 31 DAMs between S1 and S2: 8 cyanidins, 7 delphinidins, 3 malvidins, 4 pelargonidins, 2 peonidins, 3 petunidins, and 4 proanthocyanidins (**Figure 2A** and **Supplementary Table 2**). There were 28 DAMs between S2 and S3 including 7 cyanidins, 6 delphinidins, 2 malvidins, 3 pelargonidins, 2 peonidins, 3 petunidins, and 5 proanthocyanidins (**Figure 2A** and **Supplementary Table 2**). There were 14 DAMs between S3 and S4. Of these DAMs, most anthocyanins were significantly more abundant at S3 and S4 compared with S1 and S2. A number of DAMs were shared between the two comparisons: three, three, and four DAMs were shared between the S1/S2 and S2/S3, S2/S3 and S3/S4, and S1/S2 and S3/S4 comparisons, respectively (**Figure 2B**).

### Transcriptomic analysis of the *H. miconioides* sepals during development

**Figure 3:**
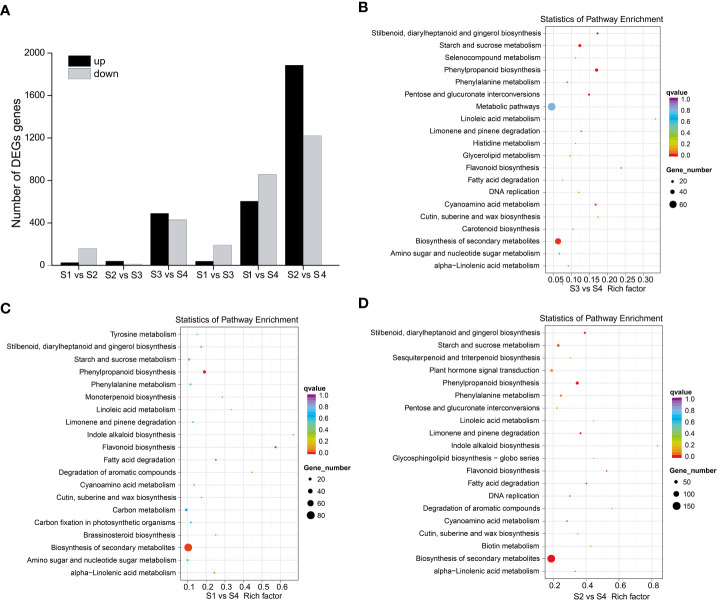
DEGs and KEGG enrichment analyses of sepal color during *Heptacodium miconioides* sepal development. **(A)** Number of up- and down-regulated DEGs in six comparisons. **(B–D)** The top 20 pathways of the DEGs based on a KEGG analysis of S3 vs. S4, S1 vs. S4, and S2 vs. S4.

To elucidate the molecular mechanisms of anthocyanin biosynthesis in the sepal of *H. miconioides*, a transcriptome analysis was performed at the four developmental stages to identify DEGs. A total of 89.69 GB of clean data were generated and the clean reads ranged from 38471686 to 63799778 for each library, with bases scoring Q30 in an average of 89.63–92.49% (**Supplementary Table 3**). The clean reads were obtained from RNA-seq raw data by filtering out uncertain reads, adapter related reads, and low-quality reads. These high-quality reads guaranteed the further gene expression analysis. Based on a |log_2_(FoldChange)| ≥1 and a *p*-value < 0.05, a total of 185 (27 up- and 158 down-regulated), 51 (40 up- and 11 down-regulated), 920 (490 up- and 430 down-regulated), 230 (39 up- and 191 down-regulated), 1460 (604 up- and 856 down-regulated), and 3106 (1886 up- and 1220 down-regulated) DEGs were identified for the S1 vs. S2, S2 vs. S3, S3 vs. S4, S1 vs. S3, S1 vs. S4, and S2 vs. S4 comparisons, respectively (**Figure 3A** and **Supplementary Figure 1**). The results indicated that the gene expression data for S2 and S3 was closer, whereas the expression profiles of S3 vs. S4, S1 vs. S4, and S2 vs. S4 were considerably different. KEGG analysis of the DEGs was conducted for the different stages and showed enrichment in many metabolic pathways including biosynthesis of secondary metabolic pathways, starch and sucrose metabolism pathways, phenylalanine metabolism pathways, flavonoid metabolism pathways, and phenylpropanoid biosynthesis pathways (**Figures 3B–D**). Of these, the last three metabolic pathways were closely associated with anthocyanin biosynthesis and were significantly enriched in the developmental stages of the sepal.

### DEG expression of anthocyanin biosynthetic structural genes

**Figure 4:**
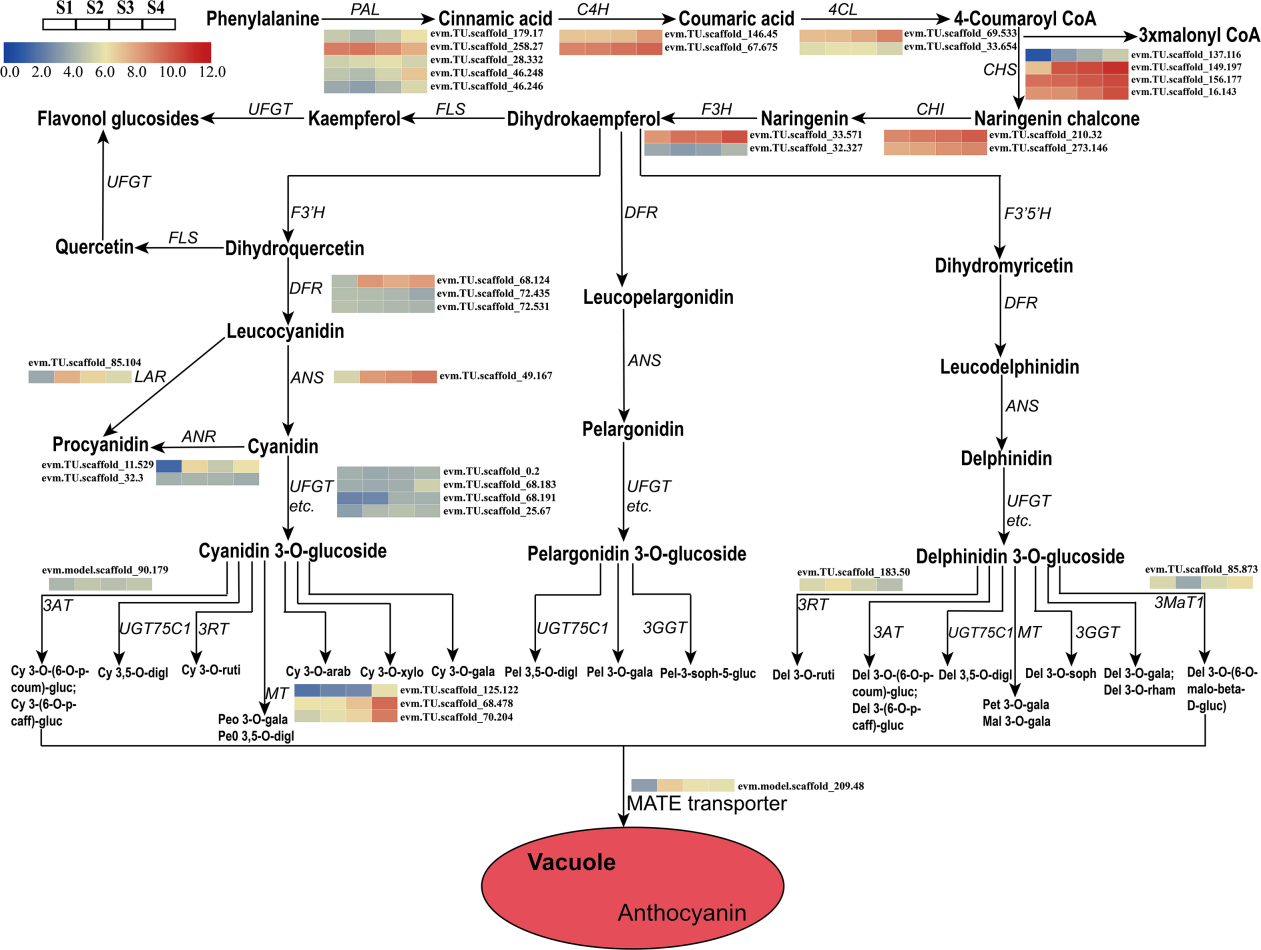
Expression analyses of structural genes involved in the pathway of anthocyanin biosynthesis at four developmental stages in the sepal were evaluated by RNA-Seq. Cy, cyanidin; Peo, Peonidin; digl, diglucoside; gluc, glucoside; coum, coumaroyl; caff, caffeoyl; ruti, rutinoside; gala, galactoside; arab, arabinoside; xylo, xyloside; Pel, Pelargonidin; soph, sophoroside; rham, rhamnoside; malo, malonyl; Pet, Petunidin; Mal, Malvidin; Del, Delphinidin.

A total of 28 candidate genes (encoding 11 key enzymes) involved in the anthocyanin biosynthetic pathway were identified based on KEGG pathway and Swiss-Prot annotations. Candidate genes were identified as showing high transcriptional activity in at least one sepal developmental stage and their expression levels were high (FPKM>10.0). These putative structural genes included *HmPAL* (5), *HmC4H* (2), *Hm4CL* (2), *HmCHS* (4), *HmCHI* (2), *HmLAR* (1), *HmF3H* (2), *HmANR* (2), *HmDFR* (3), *HmANS* (1), and *HmUFGT* (4) (**Table 1**). The expression patterns of these genes were different in the four developmental stages. A comparison of the four sepal stages revealed 15 DEGs including four *HmPAL* genes, one *HmC4H* gene, one *Hm4CL* gene, four *HmCHS* genes, one *HmLAR* gene, one *HmF3H* gene, one *HmANR* gene, one *HmDFR* gene, and one *HmANS* gene (**Supplementary Table 4**). The expression levels of three DEGs (*HmPAL*, *HmDFR*, and *HmLAR*) decreased with development and peaked during the S2 stage (**Table 1**; **Figure 4**). The other DEGs exhibited varying expression patterns; however, their expression levels were the highest during the S4 stage. In particular, one *HmANR* gene, one *HmDFR* gene, three *HmCHS* genes, and one *HmANS* gene were upregulated by more than 10-fold in the S1 stage. In addition, we also identified the glycosyltransferases (GTs) genes responsible for further glycosylation modification of anthocyanins. *HmMT* (3) and *Hm3MaT1* (1) showed a higher level of expression increased with development and peaked during the S4 stage. The expression levels of *HmRT* (3) and *HmMATE* (1) were the highest during the S2 stage.

**Table 1:** Candidates in the anthocyanin structural genes of *Heptacodium miconioides* sepal.

Gene_id	Gene_name	S1_FPKM	S2_FPKM	S3_FPKM	S4_FPKM
evm.TU.scaffold_179.17	HmPAL_1	27.65	18.07	27.73	62.38
evm.TU.scaffold_258.27	HmPAL_2	407.99	487.78	312.19	162.41
evm.TU.scaffold_28.332	HmPAL_3	32.28	39.43	49.63	34.37
evm.TU.scaffold_46.248	HmPAL_4	23.56	18.37	35.16	106.54
evm.TU.scaffold_46.246	HmPAL_5	11.86	9.23	17.43	39.21
evm.TU.scaffold_146.45	HmC4H_1	107.15	104.65	123.12	233.62
evm.TU.scaffold_67.675	HmC4H_2	368.18	397.51	536.88	694.59
evm.TU.scaffold_69.533	Hm4CL_1	120.06	126.20	178.46	373.70
evm.TU.scaffold_33.654	Hm4CL_2	45.32	50.82	50.86	35.41
evm.TU.scaffold_137.116	HmCHS_1	0.71	8.45	14.99	27.85
evm.TU.scaffold_149.197	HmCHS_2	116.29	933.34	1161.07	1943.68
evm.TU.scaffold_156.177	HmCHS_3	551.62	629.63	889.07	1160.46
evm.TU.scaffold_16.143	HmCHS_4	286.40	297.20	488.78	1054.48
evm.TU.scaffold_210.32	HmCHI_1	342.58	463.85	541.08	773.59
evm.TU.scaffold_273.146	HmCHI_2	164.73	193.04	292.88	347.57
evm.TU.scaffold_33.571	HmF3H_1	250.62	538.82	527.68	999.63
evm.TU.scaffold_32.327	HmF3H_2	11.80	8.19	9.88	16.79
evm.TU.scaffold_85.104	HmLAR_1	11.90	157.00	80.69	39.51
evm.TU.scaffold_11.529	HmANR_1	1.71	74.54	27.32	61.92
evm.TU.scaffold_32.3	HmANR_2	13.75	15.57	15.10	13.16
evm.TU.scaffold_68.124	HmDFR_1	18.80	249.28	172.73	230.77
evm.TU.scaffold_72.435	HmDFR_2	20.02	18.42	16.92	11.81
evm.TU.scaffold_72.531	HmDFR_3	21.53	18.52	17.44	15.57
evm.TU.scaffold_49.167	HmANS_1	35.49	238.50	308.90	470.60
evm.TU.scaffold_0.2	HmUFGT_1	13.83	11.67	13.23	15.88
evm.TU.scaffold_68.183	HmUFGT_2	13.11	12.10	13.52	32.37
evm.TU.scaffold_68.191	HmUFGT_3	4.47	4.72	14.53	14.43
evm.TU.scaffold_25.67	HmUFGT_4	9.38	17.74	21.54	17.46
evm.model.scaffold_90.179	Hm3AT	16.42	24.49	20.69	25.65
evm.TU.scaffold_125.122	HmMT_1	2.60	4.41	5.12	49.64
evm.TU.scaffold_68.478	HmMT_2	58.91	61.71	110.15	614.92
evm.TU.scaffold_70.204	HmMT_3	31.12	47.98	80.20	435.53
evm.TU.scaffold_183.50	Hm3RT	39.19	67.10	31.85	20.36
evm.TU.scaffold_85.873	Hm3MaT_1	38.72	11.66	38.48	66.13
evm.TU.scaffold_209.48	HmMATE	9.17	93.08	51.74	49.15

### Correlation between DEGs associated with anthocyanin biosynthesis and differentially abundant anthocyanin compounds

**Figure 5:**
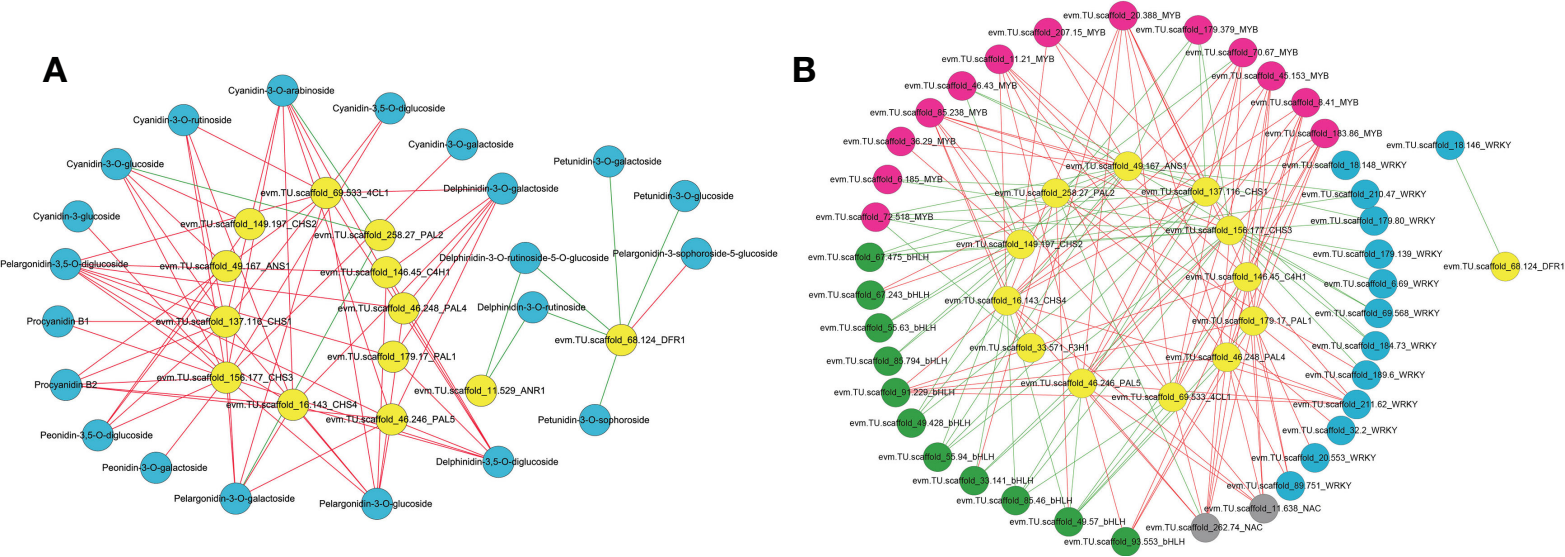
Correlation network analysis of anthocyanin structural genes with anthocyanin components, MYB, bHLH, WRKY and NAC TFs in the sepal of *Heptacodium miconioides*. **(A)** Interaction network between selected genes and anthocyanins. **(B)** Interaction network between selected genes and candidate transcription factors. The red line indicates a positive correlation and the green line indicates a negative correlation.

To identify regulatory candidate genes involved in anthocyanin biosynthesis in *H. miconioides* sepals, a network correlation analysis between the expression of 15 DEGs and the amount of 38 DAMs was conducted. All correlations between the DEGs and DAMs are shown in **Supplementary Table 5**. There were 13 differentially expressed anthocyanin structural genes that exhibited strong correlation coefficients (PCC > 0.9, *P* < 0.05) with 20 anthocyanins and their interaction networks were organized and presented in **Figure 5A**. In particular, the relative amount of cyanidin-3,5-O-diglucoside, cyanidin-3-O-galactoside, cyanidin-3-O-glucoside, proanthocyanidin B3, pelargonidin-3-O-glucoside, and peonidin-3-O-galactoside was significantly positively correlated with *HmPAL5*, *Hm4CL1*, *HmCHS1*, *HmCHS2*, *HmCHS3*, *HmCHS4*, and *HmANS1* expression (PCC > 0.9, *P* < 0.05). These anthocyanins were significantly negatively correlated with *HmPAL2* (PCC < −0.9, *P* < 0.05) (**Figure 5A**). The results indicated that these genes may represent key genes affecting anthocyanin biosynthesis in the sepal.

### Correlation between DEGs encoding TFs and differentially abundant anthocyanin compounds

To further determine the candidate TFs involved in the regulation of anthocyanin biosynthesis in sepals, we analyzed MYB, bHLH, WRKY, NAC, bZIP and HSF TFs with significant differences in expression in the sepal at each developmental stage (**Supplementary Figure 2** and **Supplementary Table 6**). After screening DEGs with FPKM <1.0 at the four sepal developmental stages, 69 TFs including 30 MYB, 14 bHLH, 15 WRKY, 5 NAC, 3 bZIP, and 2 HSF TFs remained (**Supplementary Figure 2**). A correlation network was established between the anthocyanin biosynthetic pathway genes and the 69 differential TFs. Seven previously screened anthocyanin biosynthesis genes (*PAL*, *4CL*, *C4H*, *CHS*, *ANR*, *DFR*, and *ANS*) were selected as “guide genes” to analyze co-expression correlations. Of the differentially expressed TFs, 9 MYB, 3 bHLH, 4 WRKY, and 2 NAC TFs were significantly positively correlated with anthocyanin biosynthesis genes (**Supplementary Table 7**). Subsequently, the MYB, bHLH, and WRKY TFs of *H. miconioides* were used to construct a phylogenetic tree with *A. thaliana* (**Supplementary Figures 3–5**). Several MYB, bHLH, and WRKY TFs were clustered into the subgroup of the confirmed anthocyanin biosynthesis-regulating *A. thaliana* MYB, bHLH, and WRKY families, which were also significantly correlated with anthocyanin. HmMYB114 (evm.TU.scaffold 70.67, AtMYB114) and HmMYB11 (evm.TU.scaffold 85.238, AtMYB11) were positively correlated with five (*HmPAL5*, *Hm4CL1*, *HmCHS1*, *HmCHS3*, and *HmCHS4*) and seven (*Hm4CL1*, *HmCHS1*, *HmCHS2*, *HmCHS3*, *HmCHS4*, *HmF3H1*, and *HmANS1*) anthocyanin structural genes, respectively (**Figure 5B** and **Supplementary Table 6**). HmMYB12 (evm.TU.scaffold 36.29, AtMYB12) was positively correlated with two (*HmCHS2*, *HmANS1*) structural genes. HmbHLH42 (evm.TU.scaffold 67.243, AtbHLH42/AtTT8) was positively correlated with two (*HmCHS2*, *HmANS1*) anthocyanin structural genes (**Figure 5B** and **Supplementary Table 6**). HmbHLH130 (evm.TU.scaffold_91.229, AtbHLH130) was positively correlated with five (*HmPAL1*, *HmPAL4*, *HmPAL5*, *HmC4H1*, and *HmCHS4*) anthocyanin structural genes. In contrast, HmbHLH1 (evm.TU.scaffold 49.57, AtHLH1/AtHLH2) was negatively correlated with these structural genes. HmWRKY6-1 (evm.TU.scaffold_211.62, AtWRKY6) was positively correlated with six (*HmPAL1*, *HmPAL4*, *HmPAL5*, *HmC4H1*, *Hm4CL1*, and *HmCHS4*) structural genes. HmWRKY6-2 (evm.TU.scaffold_89.751, AtWRKY6) was positively correlated with three (*HmPAL1*, *HmPAL4*, and *HmC4H1*) structural genes. In addition, there were also two NAC TFs (evm.TU.scaffold 262.74, evm.TU.scaffold 11.638) that were significantly positively correlated with several anthocyanin structural genes.

### Regulation of *HmCHS4* and *HmDFR1* promoters by candidate TFs of *H. miconioides*

**Figure 6:**
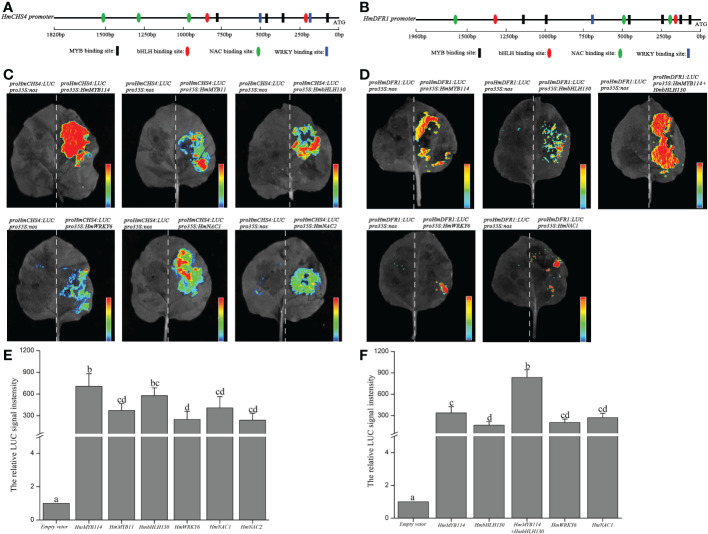
Transactivation assays of *HmCHS4* and *HmDFR1* promoters by HmMYB114, HmMYB11, HmbHLH130, HmWRKY6, HmNAC1, and HmNAC2 transcription factors. **(A, B)** Schematic overview of *HmCHS4* and *HmDFR1* promoters. **(C, D)** Imaging of luciferase enzyme activity of *HmCHS4* and *HmDFR1* promoters. Luciferase activity was detected in the tobacco leaves. The 35S-nos construct is used as a negative control. **(E, F)** The relative luciferase signal intensity of *HmCHS4* and *HmDFR1* promoters were measured in the effector and reporter vector. Values are the means ± standard deviation (SD). Data is the mean of three biological replicates. Different lowercase letters indicated significant differences at 0.05 level.

To validate the correlation between TFs and structural genes, HmMYB114, HmMYB11, HmbHLH130, HmWRKY6, HmNAC1, HmNAC2, *HmCHS4*, and *HmDFR1* were selected for subsequent functional analysis. We sequenced the upstream promoter region of *HmCHS4* (1820bp), and analyzed its cis-acting elements. Many cis-acting elements related to MYB, bHLH, WRKY, and NAC TFs were identified, including the four core MYB binding sites (5′-AACC-3′), one bHLH-binding site (5′-CATGTG-3′), one G-box (5′-CACGTG-3′), two WRKY binding sites [5′-(C/T)TGAC(T/C)-3′], and three NAC binding sites [5′-CGT(A/G)-3′] (**Figure 6A**). Analysis of the upstream promoter region of *HmDFR1* (1960bp) also found several cis-acting elements related to MYB, bHLH, WRKY, and NAC TFs, including six core MYB binding sites, two G-box binding sites, one WRKY binding site, and three NAC binding sites (**Figure 6B**). The results suggested that these TFs may have potential interaction with *HmCHS4* and *HmDFR1* promoters.

To further confirm this hypothesis, *HmCHS4* and *HmDFR1* were selected for subsequent transient expression assay in *N. benthamiana* leaves. We generated constructs including the LUC reporter gene individually driven by *HmCHS4* promoter (proHmCHS4:LUC) as reporter, constructs including HmMYB114, HmMYB11, HmbHLH130, HmWRKY6, HmNAC1, and HmNAC2 CDS driven by the cauliflower mosaic virus (CaMV35S) as effectors (pro35S:HmMYB114, pro35S:HmMYB11, pro35S:HmbHLH130, pro35S:HmWRKY6-1, pro35S:HmNAC1, and pro35S:HmNAC2, respectively), and a negative control containing only the CaMV35S promoter and the terminator (pro35S:nos). As shown in **Figure 6C**, the amount of LUC luminescence was significantly higher when each effector (pro35S:HmMYB114, pro35S:HmMYB11, pro35S:HmbHLH130, pro35S:HmWRKY6, pro35S:HmNAC1, and pro35S:HmNAC2) was co-transformed with the proHmCHS4:LUC reporter compared with the corresponding controls. The LUC luminescence was significantly higher when each effector (pro35S:HmMYB114, pro35S:HmbHLH130, pro35S:HmMYB114+HmbHLH130, pro35S:HmWRKY6, and pro35S:HmNAC1) was co-transformed with the proHmDFR1:LUC reporter (**Figures 6D, F**). These results indicated that the HmMYB114, HmbHLH130, HmWRKY6, and HmNAC1 could activate the promoters of *HmCHS4* and *HmDFR1 in vitro*.

## Discussion

### Anthocyanin identification in the *H. miconioides* sepal

Studies on color changes and anthocyanins in flowering plants have been primarily focused on petal organs and less attention has been given to sepal color changes. The molecular mechanisms of color formation in this species remain unclear. Anthocyanins are a class of water-soluble pigments present in the vacuoles of plant epidermal cells, which impart various colors to plant organs and tissues. Anthocyanin accumulation in floral organs is a key trait that affects the quality and ornamental value of plants (Zhao et al., 2012; Luo et al., 2021). In the present study, an integrated metabolome and transcriptome analysis was performed to examine the anthocyanin components and identify the genes involved in anthocyanin biosynthesis during sepal development. In recent years, the components and anthocyanin content of different plant sepals have been identified. For example, the major anthocyanin enriched in *Rhyncholaeliocattleya* Beauty Girl “KOVA” (KOVA) sepals is cyanidin O-acetylhexoside (Li et al., 2020a). Delphinidin-3-sambubioside and cyanidin-3-sambubioside are the two major anthocyanins in the calyx of *Hibiscus sabdariffa* (Hinojosa-Gómez et al., 2020). Hydrangea is a unique flower composed of sepals rather than true petals, and the main anthocyanin in the sepals is 3-O-glucosyldelphinidin (Yoshida et al., 2021). Similarly, we further identified 41 anthocyanins based on an anthocyanin-targeted metabolome assay and found that cyanidin-3,5-O-diglucoside, cyanidin-3-O-galactoside, cyanidin-3-O-glucoside, proanthocyanidin, pelargonidin-3-O-glucoside, and peonidin-3-O-galactoside are the major anthocyanins in the *H. miconioides* sepal. By analyzing the anthocyanin components in the developing sepal, we found that the content of most delphinidin, malvidin, and petunidin derivatives decreased with the development of the sepal, indicating that the delphinidin, malvidin, and petunidin components were not the main factors in the color change of the sepals. In addition, the cyanidin-3,5-O-diglucoside, cyanidin-3-O-galactoside, and cyanidin-3-O-glucoside content exhibited an increasing trend with the development of the sepal, suggesting that the high content of the cyanidin derivatives may be essential for the bright red color of the *H. miconioides* sepal. This is consistent with previous studies on ornamental plants, such as that by Shen et al. (2021), in which the red-colored flower in wintersweet was associated with cyanidin-3-O-glucoside, cyanidin-3-O-rutinoside, and cyanidin-3-O-galactoside content.

### The key candidate structural genes involved in the anthocyanin biosynthesis pathway

The regulatory mechanism for color formation is closely related to the expression of anthocyanin structural genes and regulatory genes (Forkmann and Martens, 2001; Nakatsuka et al., 2019). Previous studies have shown that several key genes and enzymes are required for anthocyanin accumulation and sepal color formation in many ornamental plants including *H. macrophylla* (Yoshida et al., 2021) and *Rhyncholaeliocattleya* Beauty Girl “KOVA” (Li et al., 2020a). However, the mechanism involved in anthocyanin biosynthesis in *H. miconioides* sepals is unclear. Anthocyanin biosynthesis is a branch of flavonoid synthesis pathway starting from phenylalanine, which involves a variety of enzymes encoded by early biosynthesis genes (*PAL*, *4CL*, *C4H*, *CHS*, *CHI*, *F3H*, *F3’H*, and *F3’5’H*) and anthocyanin biosynthesis genes (*DFR*, *ANS*, and *UFGT*) (Winkel-Shirley, 2001). In the present study, we identified 15 DEGs associated with the anthocyanin biosynthetic pathway of *H. miconioides* sepals including 11 early biosynthesis genes, 2 proanthocyanidins biosynthesis gene, and 2 anthocyanin biosynthesis genes, with almost all of them expressed at high levels during S3 and S4 compared with extremely low levels at S1–S2. These results indicate that these DEGs may play a role in the accumulation of anthocyanins at S3 to S4. Intriguingly, the expression profiles changed significantly, whereas there was no significant difference in anthocyanin product levels between S3 and S4. It is probable that the biosynthesis and accumulation of anthocyanins were closely related to environmental factors, structural genes, and TFs. The environmental factors such as temperature and light might affect the gene expression which leads to the change in anthocyanins biosynthesis and accumulation (Xue et al., 2021). Previous studies have shown that there was no significant difference in the expression of early biosynthesis genes in purple pepper and green pepper, whereas the expression of anthocyanin biosynthesis genes was significantly different (Borovsky et al., 2004; Jung et al., 2019). In addition, it was reported that the yellow color of *Paeonia lactiflora* cultivar “Huangjinlun” results from the accumulation of chalcone induced by low expression of *PlCHI* (Zhao et al., 2012). In our study, a transcriptome and metabolome association analysis results revealed that the accumulation of anthocyanins was significantly positively correlated with the expression levels of several candidate genes. *HmPAL*, *Hm4CL*, *HmC4H*, *HmCHS*, *HmF3H*, and *HmANS* expression exhibited a strong positive correlation with anthocyanin content during the four sepal stages and these expression patterns were consistent with the results of previous studies. For example, in Cymbidium hybrid flowers, the expression of *ANS* and *DFR* was closely associated with the patterns of anthocyanin accumulation (Nakatsuka et al., 2019). The expression of *CHS* and other anthocyanin genes (*UFGT2* and *DFR*) was also implicated in the accumulation of anthocyanin in mature fruits from different strawberry cultivars (Shen et al., 2021). Of these, the expression of the *HmCHS HmF3H1*, and *HmANS* genes was upregulated and highly expressed in the whole developmental stages, which contributed to the production of anthocyanin derivatives. Of note, we identified more than one anthocyanin biosynthesis-related *HmCHS* gene (four), suggesting that these genes have multiple transcripts or different gene family members. These results suggest that the high copy numbers and expression levels of key genes involved in the anthocyanidin biosynthesis pathway drive the production of these major anthocyanidin compounds in the sepal.

Colourful pigments were closely related to the biosynthesis of anthocyanins and proanthocyanidin. Proanthocyanidin are biopolymers composed primarily of epicatechin and catechin units (Dixon et al., 2005). *LAR* and *ANR* are considered two key enzymes in proanthocyanidin biosynthesis, which is in competition with the anthocyanidin formation catalysed by *ANS* and 3-glucosyltransferase (*3GT*), respectively (Pucker et al., 2020). It has been reported that the low expression levels of *LAR* and *ANR* in *Trifolium repens* leaves eventually results in a lack of proanthocyanidin biosynthesis (Ma et al., 2022). The suppression of *ANR* function caused a reduction in proanthocyanidin biosynthesis in the in *Glycine max* seed coat, with increased anthocyanin accumulation (Kovinich et al., 2012). In our study, the *HmANR1* gene was highly expressed at the S2 and S4 stages and contributed to the increase of proanthocyanidin products. This spatiotemporal pattern of proanthocyanidin might play a vital role in the growth and development of sepals and be related to potential antioxidants (Bagchi et al., 2000; Dixon et al., 2005). So far, the anthocyanidin biosynthesis pathway of mechanism in the sepal of *H. miconioides* is not clear. In this study, we discovered that cyanidin’s derivative is the main factor affecting *H. miconioides* sepal color. By analyzing the transcriptome data of four developmental sepal stages with different sepal colors, we hypothesized the putative pathway of anthocyanin biosynthesis in the sepal of *H. miconioides*. The putative pathway contains 15 protein families, including *PAL*, *4CL*, *C4H*, *CHS*, *CHI*, *F3H*, *ANS*, *LAR*, *ANR*, *UFGT*, *3MaT*, *3RT*, *3AT, MT*, and *MATE*. These results will further improve understanding of this anthocyanin biosynthesis and accumulation in *H. miconioides* sepals.

### Candidate TFs involved in anthocyanin accumulation in the *H. miconioides* sepal

In addition to structural genes, TFs also play an important role in the regulation of anthocyanin biosynthesis. In the present study, the MYB, bHLH, and other TFs, such as WRKYs, bZIPs NACs, HY5s, and ERFs were classified and identified. Previous studies have shown that the MYB-bHLH-WD40 complex represents a major class of TFs regulating the structural genes of the anthocyanin pathway including those in *A. thaliana* (Nesi et al., 2001), grapes (Deluc et al., 2006), persimmons (Akagi et al., 2009), and strawberries (Schaart et al., 2013). Thus far, several R2R3-MYB genes have been identified that control the expression of the key *CHS*, *CHI*, and *F3H* genes in early anthocyanin biosynthesis (Mehrtens et al., 2005; Stracke et al., 2007). In the present study, four candidate MYB TFs were identified from the DEG data, which exhibited a strong positive correlation with the expression of structural genes. One of these, HmMYB114 (evm.TU.scaffold_70.67), was in the same clade as MYB75, MYB90, MYB113, and MYB114 in *A. thaliana*. Previous studies demonstrated that AtMYB75, AtMYB90, AtMYB113, and AtMYB114 are involved in the MYB-bHLH-WD40 complex, which regulates the expression of anthocyanidin biosynthesis-related genes and enhances anthocyanin content (Mondal and Roy, 2018). The other two HmMYBs (evm.TU.scaffold_85.238; evm.TU.scaffold_36.29) were assigned to a large clade with AtMYB11, AtMYB12, and AtMYB111, which are associated with flavonol biosynthesis genes (*CHS*, *CHI*, *F3H*, and *FLS*) as transcriptional activators (Stracke et al., 2007; Naik et al., 2021). Both of HmMYBs are strongly expressed in *H. miconioides* sepals and are positively correlated with structural gene expression. In addition, bHLH family members frequently interact with MYB TFs to regulate anthocyanin biosynthesis through downstream gene expression (Spelt et al., 2002). The bHLH TFs can also directly bind to the promoter regions of structural genes to regulate the expression of the *CHS*, *DFR*, and *UFGT* anthocyanin genes in *Capsicum annuum* (Stommel et al., 2009). Most bHLHs involved in regulating anthocyanin biosynthesis are relatively conserved. In the present study, as shown in the phylogenetic tree, two HmbHLHs (evm.TU.scaffold_67.243; evm.TU.scaffold_49.57) were in the same clade as AtTT8, AtbHLH1, and AtbHLH2, which are involved in anthocyanin biosynthesis (Wang et al., 2019). However, TTG1 (a WD40 TF, evm.TU.scaffold_93.62) showed no significant differences at the transcriptional level at the four sepal developmental stages of *H. miconioides* in our dataset.

Aside from the MYB-bHLH-WD40 complex, other TFs have also been shown to play a role in controlling anthocyanin accumulation, such as the AP3- and AGL6-like genes (an MADS domain TF) in Cattleya hybrid “KOVA” (Li et al., 2020a), NAC56a/b (an NAC TF) in sweet potato (Wei et al., 2020), MdERF1B (an AP2/ERF TF) in apple (Zhang et al., 2018), PyWRKY26 (a WRKY TF) in pear (Li et al., 2020b), and SlHY5 (a bZIP TF) in tomato (Liu et al., 2018). Li et al. (2020a) found that the AP3- and AGL6-like MADS-box genes have multiple functions and are involved in the differential shape and color of sepals. In the present study, eight differentially expressed MADS-box TFs showed no correlation with the expression of structural genes, indicating that the color changes in the sepals of *H. miconioides* are different from that of orchids (data not show). Previous studies have shown that PyWRKY26 and PyWRKY31 regulate color formation in red-skinned pears and occurs through the interaction of PyWRKY26 and PybHLH3 co-targeting the PyMYB114 promoter (Li et al., 2020b). Of these, PyWRKY31 exhibits high homology with AtWRKY6. In the present study, two HmWRKY (evm.TU.scaffold_211.62; evm.TU.scaffold_89.751) TFs were identified that are highly homologous to AtWRKY6. Here, two HmWRKYs and two HmNACs exhibited a strong positive correlation with the expression of structural genes. Previous studies have shown that the recognition sequences of MYB, bHLH, WRKY and NAC proteins binding motif are mainly AACC, CANNTG, (C/T)TGAC(T/C) and CGT[A/G], respectively (Xiong et al., 2016; Wang et al., 2017; Luo et al., 2021). In this study, we analyzed the *HmCHS4* and *HmDFR1* promoter regions for binding sites of these TFs, indicating potential transcriptional regulation. Luciferase activity assays further confirmed that HmMYB114, HmbHLH130, HmWRKY6, and HmNAC1 significantly increased the transcriptional activity of the promoters of the structural genes *HmCHS4* and *HmDFR1* in *N. benthamiana* leaves. In addition, co-transformation with HmMYB114 and HmbHLH130 had a stronger activation effect on the *HmDFR1* promoter sequence than other TFs. The results indicated that these TFs can be potentially regarded as candidate genes for the regulation of anthocyanin biosynthesis. However, most of the differentially expressed TFs in the sepal might be connected to the development of floral organs. Future work is required to further isolate anthocyanin-related TFs from floral organs development. In this study, the development of sepal may be accompanied by changes in environmental factors such as temperature. Previous studies have shown that low temperature will increase anthocyanin accumulation during grape fruit ripening, while high temperature will lead to anthocyanin degradation (Yamane and Shibayama, 2006). The anthocyanin accumulation in sepal was separately affected by environment factors, which needed to be distinguished by further experiments.

Taken together, our study elucidated anthocyanin metabolites that contribute to the color conversion in *H. miconioides* sepals. A potential detailed pathway of anthocyanin biosynthesis in sepals of *H. miconioides* was proposed. We also showed that the altered expression of the anthocyanin pathway genes and regulatory TFs account for the difference in anthocyanin accumulation in the sepal at different developmental stages. Furthermore, the precise functional mechanism of stable transgenic requires further investigation to verify their role in regulation of anthocyanin biosynthesis.

## Data availability statement

The *H. miconioides* sepal RNA sequencing data have been submitted to NCBI under the accession no. PRJNA723161 (accession: SRX10718801; SRX10718800; SRX10718799; SRX10718798; SRX10718797; SRX10718796; SRX10718795; SRX10718794; SRX10718793; SRX10718792; SRX10718791; SRX10718790). The genome FASTA assembly and annotations files are available on FigShare at the link: https://doi.org/10.6084/m9.figshare.16803631.v1. The raw data of the metabolome analyses have been identified in MetaboLights as MTBLS6277.

## Author contributions

JML and ZXJ conceived and designed the experiments. YLL and ZSS collected the samples and performed the experiments. JYL provided technical assistance to YLL YLL wrote the draft manuscript and then ZXJ and JML revised the manuscript. All authors contributed to the article and approved the submitted version.
