# Supplementary material for: Integrated transcriptomics and metabolomics analysis provide insight into anthocyanin biosynthesis for sepal color formation in Heptacodium miconioides
Source: Front Plant Sci. 2023 Feb 20;14:1044581. doi: 10.3389/fpls.2023.1044581 (PMC9987713; doi:10.3389/fpls.2023.1044581)
Supplement: Supplementary file 1 [file DataSheet_1.docx]

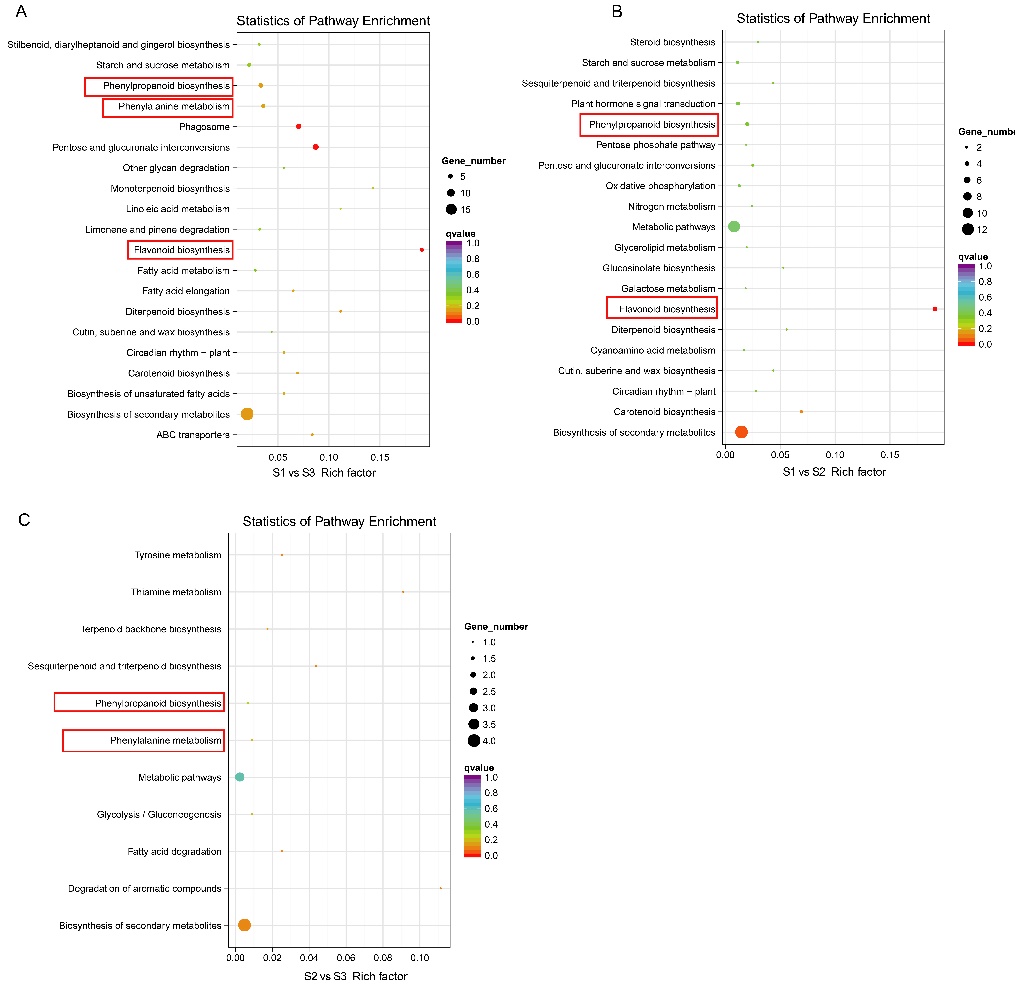


Fig. S1 The top 20 pathways of the DEGs based on KEGG in S1 vs S3, S1 vs S2, and S2 vs S3, respectively (A-C). The red rectangle highlights the pathway associated with anthocyanin metabolism.


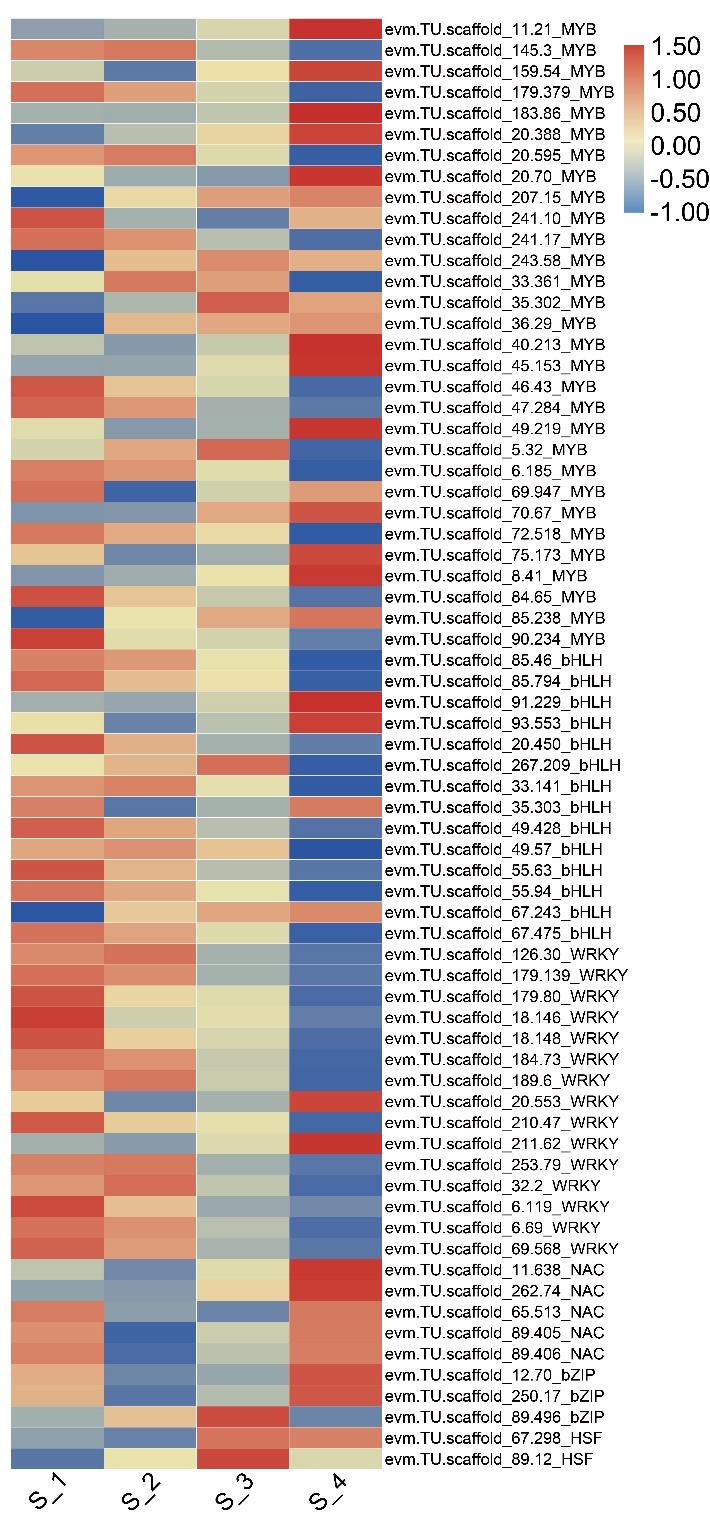


Fig. S2. Heat map showing the 69 differentially expressed TFs (including 30 MYBs, 14 bHLHs, 15 WRKYs, 5 NACs, 3 bZIPs, and 2 HSFs) between the four sepals at each stage. Cell colors correspond to the log2 magnitude of the difference in expression level log_2_(FPKM+1): red cells indicate upregulation, whereas blue cells indicate downregulation.


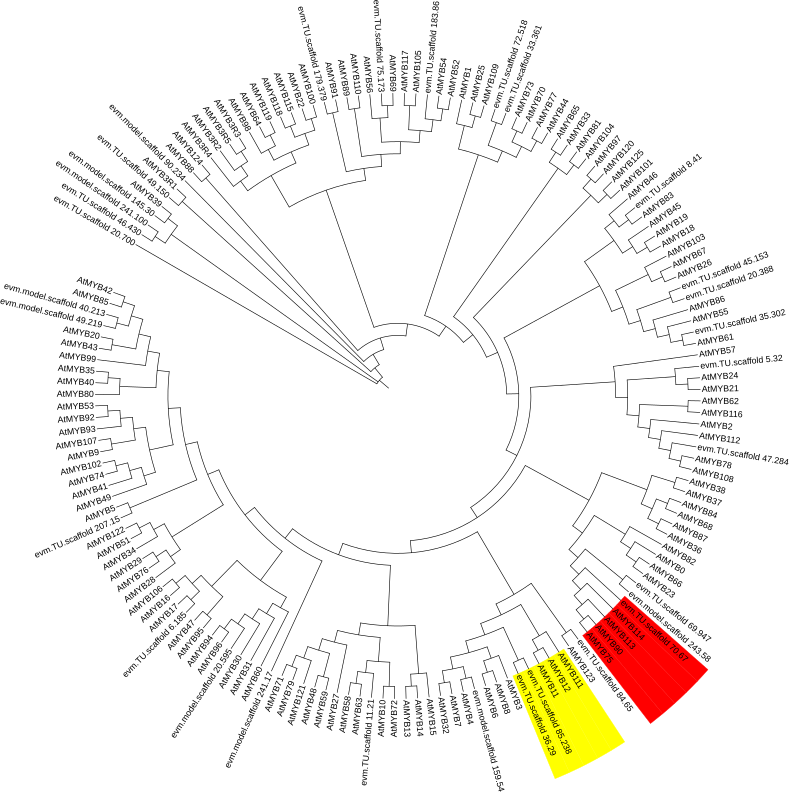


Fig. S3. Phylogenetic analysis of differentially expressed MYB transcription factors in sepals of *Heptacodium miconioides* and *Arabidopsis thaliana* MYB transcription factors. Different color boxes represent the clustering results of different transcription factors.


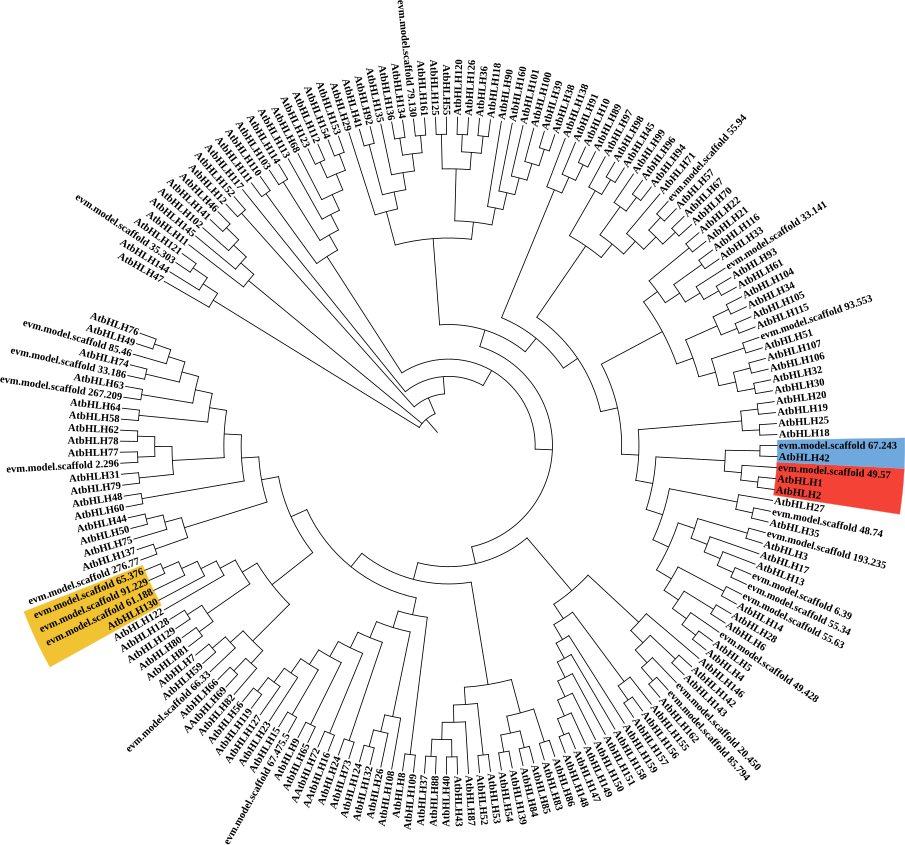


Fig. S4. Phylogenetic analysis of differentially expressed bHLH transcription factors in sepals of *Heptacodium miconioides* and *Arabidopsis thaliana* bHLH transcription factors. Different color boxes represent the clustering results of different transcription factors.


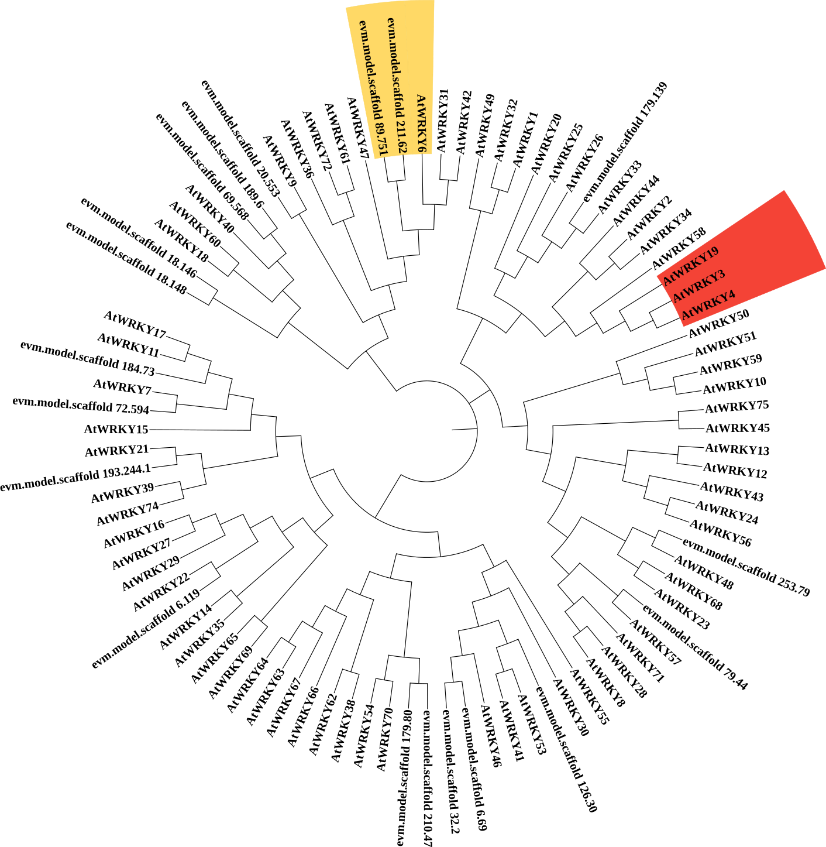


Fig S5. Phylogenetic analysis of differentially expressed WRKY transcription factors in sepals of *Heptacodium miconioides* and *Arabidopsis thaliana* MYB transcription factors. Different color boxes represent the clustering results of different transcription factors.
